# Virulence gene profiling of porcine *Pasteurella multocida* isolates of Assam

**DOI:** 10.14202/vetworld.2018.348-354

**Published:** 2018-03-21

**Authors:** L. Babita Devi, Durlav Prasad Bora, S. K. Das, R. K. Sharma, S. Mukherjee, R. A. Hazarika

**Affiliations:** 1 KVK Churachandpur, ICAR Manipur Centre, Imphal, Manipur, India; 2 Department of Microbiology, College of Veterinary Science, AAU, Khanapara, Guwahati - 781 022, Assam, India; 3 Department of Veterinary Epidemiology and Preventive Medicine, CVSc, CAU, Aizawl, Mizoram, India; 4 Department of Veterinary Public Health, College of Veterinary Science, AAU, Khanapara, Guwahati - 781 022, Assam, India

**Keywords:** capsular type, *Pasteurella multocida*, porcine, virulence-associated genes

## Abstract

**Aim:**

The present study was conducted to detect and identify the virulence genes in *Pasteurella multocida* isolates of porcine origin from Assam.

**Materials and Methods:**

A total of 21 porcine *P. multocida* isolates were subjected to capsular typing and detection of virulence-associated genes (*pfhA, tbpA, hgbB, toxA, oma87, ompH*, and *nanB*) using various polymerase chain reaction (PCR) methods reported elsewhere. Further, pathogenicity of the porcine isolates of *P. multocida* was studied in mice. For each strain of *P. multocida* selected for pathogenicity trial, the group of mice was injected intraperitoneally (i/p) with 0.1 ml of the inoculum prepared from respective field isolates, containing 10^9^ organisms per ml.

**Results:**

Capsular typing of the isolates by multiplex PCR showed two capsular types, type A (66.66%) and type D (33.33%). All the isolates were positive for outer membrane protein genes, *oma87* and *ompH* genes. Iron acquisition genes, *tbpA* and *hgbB*, were detected in 14.28% and 19.04% of the isolates. The dermonecrotoxin encoding gene, *toxA*, was present in 23.80% of the isolates. Filamentous hemagglutinin encoding gene, *pfhA*, was detected in 28.57%. The virulence gene distribution pattern of the isolates indicates the important role of the genes in disease pathogenesis.

**Conclusion:**

From the present study, it can be concluded that *toxA* gene is an important marker gene for defining the pathogenic potential of *P. multocida* strains in swine.

## Introduction

*Pasteurella multocida* belonging to family Pasteurellaceae is a ubiquitous organism affecting multiple host species, thus causing several diseases such as hemorrhagic septicemia in cattle and buffalo, enzootic bronchopneumonia in cattle, sheep, and goats, atrophic rhinitis in swine, fowl cholera in poultry, and snuffles in rabbits [1,2]. It is one of the most fascinating Gram-negative, opportunistic animals and human pathogens with worldwide distribution.

The organism is grouped into 5 capsular serogroups (A, B, D, E, and F) with host specificity and disease induction [3]. Both toxigenic and non-toxigenic strains of serogroups A and D are associated with diseases in swine [4]. The pathogenicity of *P. multocida* is associated with various virulence factors which include diverse adhesions, dermonecrotic toxin, iron acquisition proteins, sialidases, and outer membrane proteins [3,5-7]. These virulence factors help in colonization and invasion of the host, avoid host defense mechanisms, injury to host tissues, and stimulate host inflammatory response. The association of virulence factors with specific serogroups of *P. multocida* and its disease status in animals was also reported by Ewers *et al*. [8]. Since the pathogenic behavior of *P. multocida* could be predicted both by the virulence factors and the serogroups, evaluation of these virulence factors is important.

The present study investigates the distribution pattern of virulence-associated genes (VGAs) in *P. multocida* isolates of porcine origin.

## Materials and Methods

### Ethical approval

Ethical approval for the study was obtained from IAEC, Assam Agricultural University (AAU), Khanapara campus vide approval No. 770/ac/CPCSEA/FVSc/AAU/IAEC/10-11/79 dated 09.09.2011.

### Source of *P. multocida* isolates

Twenty-one *P. multocida* isolates maintained in the ICAR Network Project on Hemorrhagic Septicemia, Department of Microbiology, College of Veterinary Science, Assam Agricultural University, Khanapara, Guwahati, were used for the study. The reference strain (P_52_) was obtained from the Division of Bacteriology and Mycology, ICAR-Indian Veterinary Research Institute, Izatnagar, Bareilly, Uttar Pradesh.

### Revival and confirmation of *P. multocida* isolates

The isolates were reconfirmed by following standard bacteriological techniques and by *P. multocida* species-specific polymerase chain reaction (PM-PCR) as per the method described by Townsend *et al*. [9] using specific primer pairs (Table-1) [7,9-13]. PCR was done in 25 μl reaction mixture by mixing 3.0 μl DNA template with 12.5 μl master mix (2X, Qiagen, Germany) and forward and reverse primers (10 pmol each). The PCR amplification was performed in a thermocycler (Applied Biosystems, USA) using the thermal conditions as initial denaturation at 94°C for 4 min, 35 cycles of 94°C for 45 s, 55°C for 45 s, 72°C for 45 s, followed by final extension at 72°C for 6 min.

**Table-1:** Sequences of the oligonucleotides used in the *P. multocida* multiplex capsular and virulence-associated genes typing assay of *P. multocida*.

Gene	Primer	Sequence (5’-3’)	Amplicon size	References
*KMT1*	KMT1T7 Fwd	ATCCGCTATTTACCCAGTGG	460 bp	Townsend *et al*. [9]
	KMT1SP6 Rev	GCTGTAAACGAACTCGCCAC		
*hyaD-hyaC*	CAPA Fwd	TGCCAAAATCGCAGTCAG	1044 bp	Townsend *et al*. [10]
	CAPA Rev	TTGCCATCATTGTCAGTG		
*bcbD*	CAPB Fwd	CATTTATCCAAGCTCCACC	760 bp	Townsend *et al*. [10]
	CAPB Rev	GCCCGAGAGTTTCAATCC		
*dcbF*	CAPD Fwd	TTACAAAAGAAAGACTAGGAGCCC	657 bp	Townsend *et al*. [10]
	CAPD Rev	CATCTACCCACTCAACCATATCAG		
*toxA*	Forward	TCT TAG ATG AGC GAC AAG G	846 bp	Shayegh *et al*. [11]
	Reverse	GAA TGC CAC ACC TCT ATA G		
*hgbB*	Forward	TCT TTG AGT ACG GCT TGA C	540 bp	Shayegh *et al*. [11]
	Reverse	CTT ACG TCA GTA ACA CTC G		
*tbpA*	Forward	TGG TTG GAA ACG GTA AAG C	728 bp	Shayegh *et al*. [11]
	Reverse	TAA CGT GTA CGG AAA AGC C		
*pfhA*	Forward	AGC TGA TCA AGT GGT GAA C	275 bp	Shayegh *et al*. [11]
	Reverse	TGG TAC ATT GGT GAA TGC TG		
*nanB*	Forward	CAT TGC ACC TAA CAC CTC T	555 bp	Tang *et al*. [12]; Ewer *et al*. [7]
	Reverse	GGA CAC TGA TTG CCC TGA A		
*Oma87*	Forward	GGC AGC GAG CAA CAG ATA ACG	838 bp	Tang *et al*. [12]; Ewer *et al*. [7]
	Reverse	TGT TCG TCA AAT GTC GGG TGA		
*ompH*	Forward	GCG TTT CAT TCA AAG CAT CTC	1000 bp	Luo *et al*. [13]
	Reverse	ATG ACC GCG TAA CGA CTT TC		

P. multocida=Pasteurella multocida

### Capsular typing

Capsular PCR typing for all the isolates was done using a multiplex PCR as per the method described by Townsend *et al*. [10] with the reaction condition as illustrated in Table-2 [7,11].

**Table-2:** Thermal cycling condition for the detection of virulence-associated genes of *P. multocida*.

PCR steps	Gene						

	*toxA*	*tbpA*	*hgbB*	*pfhA*	*ompH*	*oma87*	*nanB*
Initial denaturation	95°C 5 min	95°C 5 min	95°C 5 min	95°C 5 min	94°C 3 min	94°C 3 min	94°C 3 min
Denaturation	94°C 45 s	94°C 45 s	94°C 45 s	94°C 45 s	94°C 30 s	94°C 30 s	94°C 30 s
Annealing	54°C 50 s	54°C 50 s	54°C 50 s	54°C 50 s	57°C 30 s	55°C 30 s	56°C 30 s
Extension	72°C 50 s	72°C 50 s	72° C 50 s	72°C 50 s	72°C 60 s	72°C 60 s	72°C 45 s
Number of cycles	35	35	35	35	25	25	30
Final extension	72°C 10 min						
Hold	4°C						
References	Shayegh *et al*. [11]	Shayegh *et al*. [11]	Shayegh *et al*. [11]	Shayegh *et al*. [11]	Ewer *et al*. [7]	Ewer *et al*. [7]	Ewer *et al*. [7]

*P. multocida=Pasteurella multocida*, PCR=Polymerase chain reaction

### Virulence gene detection

*P. multocida* isolates were tested for the presence of various VGAs, for example, *pfhA, tbpA, hgbB, toxA, oma87, ompH*, and *nanB* by simplex PCR as per the method of Ewers *et al*. [8], using specific primers (Table-1) with the standard thermal conditions (Table-2). The amplified products were electrophoresed in 1.5% agarose gel in 1X tris acetate EDTA buffer at 60 V for 1 h with ethidium bromide stain and visualized with ultraviolet light by Gel Documentation System (Kodak, Biostep, Germany).

### Pathogenicity study of *P. multocida* isolates

Pathogenicity of the porcine isolates of *P. multocida* was studied in mice following the method described by Curtis [14] with slight modification. For each strain of *P. multocida* selected for pathogenicity trial, the group of mice was injected intraperitoneally (i/p) with 0.1 ml of the inoculum prepared from respective field isolates, containing 10^9^ organisms per ml [15].

## Results

**Figure-1:**
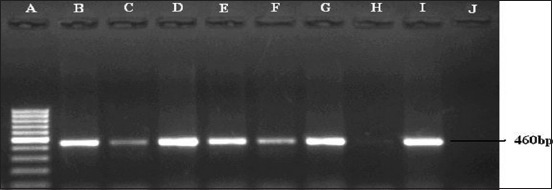
*Pasteurella multocida* species-specific polymerase chain reaction for the detection of *KMT1* gene (460 bp) of *P. multocida*. Lane A: 100 bp DNA Ladder. Lane B to H: Samples positive for *kmt* gene. Lane J: Negative control.

All the bacterial isolates were revived in blood agar. The small, smooth, circular, glistening, and dewdrop-like colonies with a very characteristic odor and non-hemolytic colonies on blood agar plate (data not shown) were found to be Gram-negative coccobacilli and identified as *P. multocida*. The organisms were further confirmed based on specific amplification of *KMT1* gene by PCR yielding an expected product size of 460 bp (Figure-1) which was detected in all the isolates.

**Figure-2:**
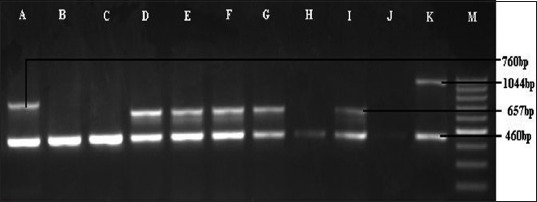
Multiplex-polymerase chain reaction for capsular typing of *Pasteurella multocida*. Lane A: Reference strain (P_52_). Lane D, E, F, G, and I: Sample positive for cap D. Lane K: Sample positive for cap A. Lane M: 100 bp DNA Ladder.

On capsular PCR typing, of 21 *P. multocida* isolates, serogroup D-specific gene (657 bp, Figure-2) was detected in seven (33.33%) isolates, while serogroup A-specific gene (1044 bp, Figure-2) was detected in 14 (66.66%) isolates. The reference strain gave an amplified product of 760 bp band size corresponding to capsular type B.

**Figure-3:**
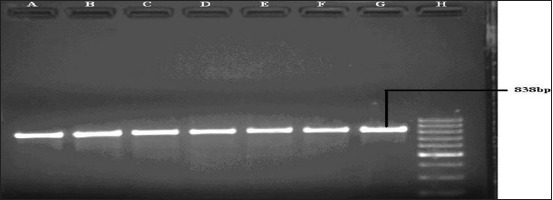
Polymerase chain reaction for the detection of *oma87*gene (838 bp) of *Pasteurella multocida*. Lane A to G: Samples positive for *oma87* gene. Lane H: 100bp DNA Ladder.

**Figure-4:**
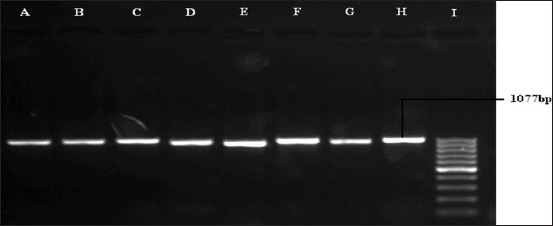
Polymerase chain reaction for the detection of *ompH* gene (1077 bp) of *Pasteurella multocida*. Lane A to H: Samples positive for *ompH* gene, Lane I: 100 bp DNA Ladder.

**Figure-5:**
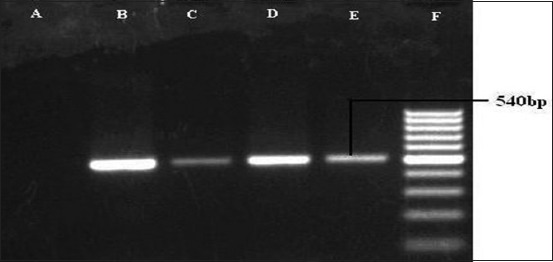
Polymerase chain reaction for the detection of *hgbB* gene (540 bp) of *Pasteurella multocida*. Lane A: Samples negative for *hgbB* gene. Lane B, C, D, and E: Samples positive for *hgbB* gene. Lane F: 100 bp DNA Ladder.

**Figure-6:**
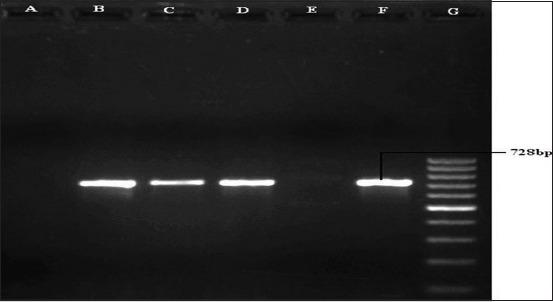
Polymerase chain reaction for the detection of *tbpA* gene (728 bp) of *Pasteurella multocida*. Lane B, C, D, and F: Samples positive for *tbpA* gene. Lane A and E: Samples negative for *tbpA* gene. Lane G: 100 bp DNA Ladder.

On virulence gene detection PCR (Table-3), it was observed that the outer membrane genes (*oma87* and *ompH*) were found to be present in all the 21 isolates of porcine origin used in the present study and the reference strain giving expected band size of 838 and 1077 bp, respectively (Figures-3 and 4). The hemoglobin binding gene (*hgbB*, Figure-5) was found in four (57.14%) isolates of serotype D only, while *tbpA* (Figure-6) gene was detected in four (21.42%) serotype A isolates only.

**Table-3:** Virulence-associated gene detection in different serotypes of swine *Pasteurella multocida* by PCR and their pathogenicity in mice.

S. No.	Isolate No.	Serotype	Virulence genes							Pathogenicity

			*toxA*	*nanB*	*tbpA*	*pfhA*	*hgbB*	*oma87*	*ompH*	
1	P2	A						+	+	(83.33)
2	P3	A						+	+	
3	P4	A						+	+	(66.66)
4	P6	A						+	+	(83.33)
5	P16	A						+	+	
6	P19	A						+	+	
7	P20	A						+	+	
8	P22	A						+	+	(50)
9	P5	A				+		+	+	(83.33)
10	P7	A				+		+	+	
11	P13	A				+		+	+	
12	P14	A	+		+	+		+	+	(100)
13	P18	A	+		+	+		+	+	(83.33)
14	P10	A	+		+			+	+	(100)
Sub total		14	3 (21.42)	0	3 (21.42)	5 (35.71)	0	14 (100)	14 (100)	8 (57.14)
15	P1	D						+	+	
16	P9	D						+	+	
17	P15	D	+	+			+	+	+	(100)
18	P17	D					+	+	+	
19	P21	D					+	+	+	(50)
20	P8	D		+				+	+	(33.33)
21	P11	D	+			+	+	+	+	(100)
Sub total		7	2 (28.57)	2 (28.57)	0	1 (14.28)	4 (57.14)	7 (100)	7 (100)	4 (57.14)
22	P12	B (P_52_)	0	0	1	1	0	1	1	(100)
Grand total		22	5 (22.72)	2 (9.09)	4 (18.18)	7 (31.81)	4 (18.18)	22 (100)	22 (100)	13 (59.09)

PCR=Polymerase chain reaction

**Figure-7:**
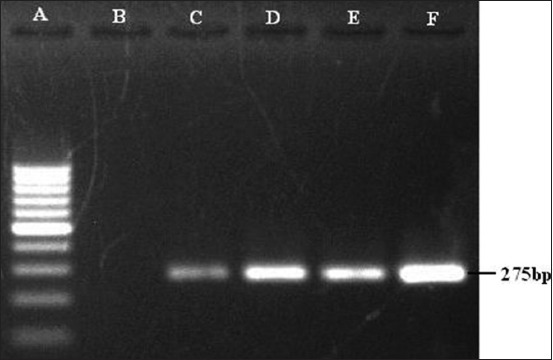
Polymerase chain reaction for the detection of *pfhA* gene (275 bp) of *Pasteurella multocida*. Lane A: 100 bp DNA Ladder, Lane B: Samples negative for *pfhA* gene, Lane C to F: Samples positive for *pfhA* gene.

**Figure-8:**
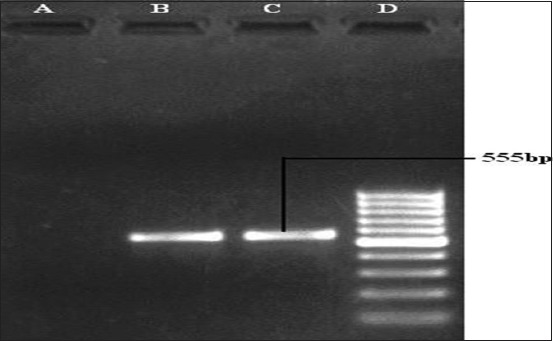
Polymerase chain reaction for the detection of *nanB* gene (555 bp) of *Pasteurella multocida*. Lane A: Samples negative for *nanB* gene. Lane B and C: Samples positive for *nanB* gene. Lane D: 100 bp DNA Ladder.

On amplification of filamentous hemagglutinin gene (*pfhA*), of the 21 isolates, six exhibited the presence of the same by yielding an expected amplicon size of 275 bp (Figure-7), of which 5 isolates belong to serotype A (35.71%) and 1 to serotype D (14.28%). The sialidase coding gene (*nanB*) was detected only in 2 (28.57%) isolates of serotype D (Figure-8).

**Figure-9:**
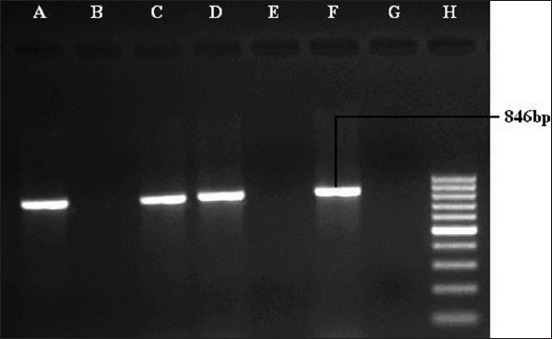
Polymerase chain reaction for the detection of *toxA* gene (846 bp) of *Pasteurella multocida*. Lane A, C, D, and F: Samples positive for *toxA* gene. Lane B, E, and G: Samples negative for *toxA* gene. Lane H: 100 bp DNA Ladder.

Toxigenic gene (*toxA*) was detected in five isolates, of which three were serotype A (21.42%), while 2 isolates of serotype D (28.57%). *toxA* gene was absent in the reference strain P_52_ strain (Figure-9).

## Discussion

The present paper describes as the first report of virulence gene profiles of porcine *P. multocida* from Assam, India. Pasteurellosis is a common disease of pigs worldwide with specific serotype and pathotype associated with the respiratory disease [16-18]. However, the distribution pattern of the serotypes and pathotypes can vary considerably from region to region and over time in a given region [12,19]. The present study results in the detection of more percentage of serotype A than serotype D. The isolates used in the present study were mainly from cases of the respiratory syndrome. A higher percentage of serotype A than serotype D in *P. multocida* isolates from indigenous pigs in central Kalorey *et al*. [20] and Northeast India [19,21] have been reported.

Variable distribution of the virulence genes was observed among the serotypes of *P. multocida* during the present study (Table-3). However, no correlation could be established with respect to the presence of virulence gene and serotypes of the Wide spread. Widespread distribution of *hgbB* gene among the porcine *P. multocida* strains and its regular detection in capD strains compared to capA strains of *P. multocida* was also reported [7,22]. On the contrary to the present detection of *tbpA* gene, Ewers *et al*. [7] could detect the gene exclusively in bovine, sheep, and buffalo isolates and not in *P. multocida* pig strains. The present observation of 18.18% positive strains of *P. multocida isolates* possessing the *tbpA* gene could be probably due to interspecies transmission of *tbpA* gene-positive *P. multocida*, as also suggested by Kumar *et al*. [23]. However, further study needs to be conducted before giving conclusive remark on interspecies transmission.

The present finding of low percent positivity of *pfhA* gene in strains of serogroup D compared with serogroup A also supports the observations of association of filamentous hemagglutinin gene *pfhA* with serogroups A, B, E, and F [7,12,22].

The occurrence of OMP gene in porcine strains of *P. multocida* and its equal distribution among capsular serogroups (type A, type D, and other serotypes) was also reported [7,12,22]. *P. multocida* OMPs have been identified as potent immunogens [24] as reported by Rajkhowa *et al*. [25] and play a significant role in the pathogenesis of pasteurellosis [26]. Hazarika *et al*. [27] also reported that the sonicated and bacterin vaccines prepared from pig strains of *P. multocida* conferred 100% protection against homologous as well as heterologous strains compared to 66.66 and 86.66% protection with vaccine prepared from reference strain (P_52_). From the present study, it can be opined that the OMP gene has a great role in pathogenesis of *P. multocida* infection irrespective of the serotypes. Considering the immunogenic potential of the OMPs, both the genes can be explored in the development of a suitable vaccine against swine pasteurellosis. However, before giving any conclusive remarks on distribution and its applicability as vaccine candidate, a detailed study will be required involving a large number of isolates recovered from pigs of different parts of the Northeastern region.

The 13 (59.09%) pathogenic isolates were found to possess different combinations of virulence genes. Pathogenic serotype A isolates with different virulence gene combinations produce 83.33-100% mortality in inoculated mice when compared to isolates with the presence of only OMP genes (50-83.33%). Similarly, serotype D isolates with different virulence gene combinations produce 33.33-100% mortality in inoculated mice. No reports could be traced out from the available literature with respect to the correlation between presence virulence gene in the *P. multocida* isolates and their pathogenicity in mice. However, it can be opined from the present observation that OMP alone is not responsible for pathogenesis of the disease and association of other VGAs with OMP gene might play a key role in pathogenesis. The present study indicates that *toxA* gene plays an important role in pathogenesis of *P. multocida* in pigs, which is supported by the findings of many workers [28-30]. Among the other virulence genes, *tbpA* gene was found to be closely associated with pathogenesis of the *P. multocida*. Contrary to the present findings, Ewers *et al*. [7] reported the presence of *tbpA* gene only in bovine strains of *P. multocida* and could not detect in porcine strains. Although they observed a significant association between *pfhA* and *toxA* with clinically diseased swine, *toxA* alone was found to be associated with the disease status independently. A perusal of the literature reveals that there is no report on the detection of *tbpA* gene from porcine strains, the detection of the gene in highly pathogenic porcine isolate is an important finding, and its role in pathogenesis needs further investigation. During the present study, some of the *P. multocida* isolates could not produce mortality in mice following inoculation with *P. multocida* isolate having virulence gene, either alone or in combination. This might be due to repeated subculturing of the isolates in laboratory media that result in the suppression of gene function or due to gene mutation, resulting in non-expression of the genes *in vivo* [31,32]. Detection of a high proportion of toxigenic capsular type A *P. multocida* from pigs was also reported by other workers [11,20,30,33]. Detection of toxin gene in both the type A and D isolates of *P. multocida* of the region in the present study indicates its important role in the disease pathogenesis mechanism.

## Conclusion

From the present study, it can be concluded that *toxA* gene is an important marker gene for defining the pathogenic potential of *P. multocida* strains in swine. However, other virulence genes are also found to be distributed well among pathogenic strains of *P. multocida*. Among the other virulence genes, *tbpA* gene was found to be closely associated with the pathogenesis of the *P. multocida*. The association of the gene in disease producing mechanism needs further evaluation.

## Authors’ Contributions

This study was a part of LBD’s research work during his PhD program. LBD carried out the experiment. SKD, RKS, and DPB designed the experiment. SKD, RKS, DPB, SM, and RAH provided necessary guidelines. DPB drafted the final manuscript. All authors have read and approved the final manuscript.
